# A Knockout of Poly(ADP-Ribose) Polymerase 1 in a Human Cell Line: An Influence on Base Excision Repair Reactions in Cellular Extracts

**DOI:** 10.3390/cells13040302

**Published:** 2024-02-06

**Authors:** Svetlana N. Khodyreva, Ekaterina S. Ilina, Nadezhda S. Dyrkheeva, Alina S. Kochetkova, Alexandra A. Yamskikh, Ekaterina A. Maltseva, Anastasia A. Malakhova, Sergey P. Medvedev, Suren M. Zakian, Olga I. Lavrik

**Affiliations:** 1 Institute of Chemical Biology and Fundamental Medicine, Siberian Branch of Russian Academy of Sciences, 8 Akad. Lavrentyeva Ave., Novosibirsk 630090, Russia; katya.plekhanova@gmail.com (E.S.I.); dyrkheeva.n.s@gmail.com (N.S.D.); kochetkovaalina96@gmail.com (A.S.K.); a.yamskikh@g.nsu.ru (A.A.Y.); 060179@mail.ru (E.A.M.); amal@bionet.nsc.ru (A.A.M.); medvedev@bionet.nsc.ru (S.P.M.); zakian@bionet.nsc.ru (S.M.Z.); 2 Faculty of Natural Sciences, Novosibirsk State University, 2 Pirogova Str., Novosibirsk 630090, Russia; 3 Federal Research Center Institute of Cytology and Genetics, Siberian Branch of Russian Academy of Sciences, 10 Akad. Lavrentyeva Ave., Novosibirsk 630090, Russia

**Keywords:** base excision repair enzymatic activity, CRISPR/Cas9, mRNA, poly(ADP-ribose) polymerase 1, poly(ADP-ribosyl)ation

## Abstract

Base excision repair (BER) is the predominant pathway for the removal of most forms of hydrolytic, oxidative, and alkylative DNA lesions. The precise functioning of BER is achieved via the regulation of each step by regulatory/accessory proteins, with the most important of them being poly(ADP-ribose) polymerase 1 (PARP1). PARP1′s regulatory functions extend to many cellular processes including the regulation of mRNA stability and decay. PARP1 can therefore affect BER both at the level of BER proteins and at the level of their mRNAs. Systematic data on how the PARP1 content affects the activities of key BER proteins and the levels of their mRNAs in human cells are extremely limited. In this study, a CRISPR/Cas9-based technique was used to knock out the *PARP1* gene in the human HEK 293FT line. The obtained cell clones with the putative PARP1 deletion were characterized by several approaches including PCR analysis of deletions in genomic DNA, Sanger sequencing of genomic DNA, quantitative PCR analysis of PARP1 mRNA, Western blot analysis of whole-cell-extract (WCE) proteins with anti-PARP1 antibodies, and PAR synthesis in WCEs. A quantitative PCR analysis of mRNAs coding for BER-related proteins—PARP2, uracil DNA glycosylase 2, apurinic/apyrimidinic endonuclease 1, DNA polymerase β, DNA ligase III, and XRCC1—did not reveal a notable influence of the PARP1 knockout. The corresponding WCE catalytic activities evaluated in parallel did not differ significantly between the mutant and parental cell lines. No noticeable effect of poly(ADP-ribose) synthesis on the activity of the above WCE enzymes was revealed either.

## 1. Introduction

Poly(ADP-ribose) polymerase 1 (PARP1), the best-known member of the ADP-ribosyltransferase family, acts as a prime sensor of DNA strand breaks; it becomes catalytically active upon binding to broken DNA. PARP1 catalyzes the formation of poly(ADP-ribose) (PAR). This process, known as PARylation, leads to the recruitment and activation of various DNA repair factors [1,2,3,4,5,6,7,8,9,10,11,12]. PARP1 is a critical sensor in (and regulator of) the DNA damage response, primarily through involvement in the base excision repair (BER) pathway. Furthermore, PARP1 is implicated in other DNA repair processes, including nucleotide excision repair, homologous recombination, and nonhomologous end joining, possibly indicating its broader participation in the maintenance of genome stability. In addition, PARP1 is involved in other cellular processes such as transcriptional regulation, chromatin remodeling, and cell death [1,2,3,4,5,6,7,8,9,10,11,12,13,14].

Base excision repair (BER) is one of the most significant DNA repair systems, since its actions are aimed at correcting the damage caused by endogenous and exogenous factors. DNA lesions, repaired by the BER system, are non-bulky lesions that do not cause a significant change in the DNA helix [15,16,17,18,19,20,21,22].

The BER pathway consists of several consecutive steps: excision, where an aberrant base is detached from the DNA backbone; incision of the phosphodiester backbone at the abasic site; gap processing that includes editing of the DNA termini, when necessary; followed by dNMP insertion and the ligation of nicks, where the DNA backbone is restored. BER is mainly initiated by one of damage-specific DNA glycosylases and can proceed either a short-patch (SP) or long-patch (LP) pathway with the replacement of one or several nucleotides, respectively [15,16,17,18,19,20,21,22]. Furthermore, this type of DNA repair comprises several minor pathways: single-strand break repair, which starts from a DNA intermediate with the already cleaved sugar-phosphate backbone of DNA [23] and nucleotide incision repair, where apurinic/apyrimidinic (AP) endonuclease 1 (APE1) initiates the DNA glycosylase–independent repair of some types of oxidized bases [24].

Data on the role of PARP1 in BER that have accumulated to date are controversial: there is evidence of positive and negative influences or a lack of influence on this process, and the predominant number of results were obtained in PARP1 knockout mouses and their cells [25,26,27,28,29,30,31,32]. In this regard, studying the effect of reduced amounts of PARP1 or its absence on the BER process in human cells seems to be an important task. This particularly applies to cells of non-cancerous origin in the absence of DNA-damaging agents. Positive influences of PARP 1 and PARP2 PARylation on the BER reactions have been demonstrated by us earlier on linear and nucleosome-arranged DNA [30,31]. Recently [32], using extracts and RNA samples obtained from the parental HEK293T cell line (HEK293T WT) and its derivative HEK293T/P1-KD cell line (featuring PARP1 under expression caused by a corresponding shRNA: a PARP1 knockdown, HEK293T/P1-KD), we assessed, through qPCR, the levels of mRNA coding for some BER proteins taking part in catalysis and regulation in the SP BER pathway: PARP1, APE1, uracil DNA glycosylase (UNG2), DNA polymerase β (POLβ), DNA ligase III (LIG3), XRCC1, and Poly(ADP-ribose)polymerase 2 (PARP2). The levels of mRNA encoding PARP1 and the amount of the PARP1 protein were roughly halved in HEK293T/P1-KD cells as compared with the parental cells. The PARP1 amount in the cells did not significantly influence mRNA levels of *UNG2*, *APE1*, *POLβ*, *LIG3*, and *XRCC1*. The catalytic activities of the BER enzymes in whole-cell extracts (WCEs), as evaluated using specific DNA substrates, also did not differ significantly between HEK293T WT and HEK293T/P1-KD cells. Under conditions of PAR synthesis, no significant change in the efficiency of the reactions catalyzed by UNG2, APE1, POLβ, and LIG3 was observed either.

Later, we evaluated the effect of a PARP1 knockout on the expression of some BER-related genes in the HEK293A (HEK293A/P1-KO) cell line obtained by the CRISPR/Cas9 technique [33,34]. Data from PCR analyses indicate a homozygous deletion in the *PARP1* gene [33]. Both *PARP1* mRNA in cells and the amount of the PARP1 protein in a WCE were barely detectable. Transcriptomic analysis revealed that among differentially expressed genes in HEK293A/P1-KO cells—compared with wild-type HEK293A cells—several enzymes involved in LP BER are downregulated by the knockout of PARP1 [34].

In the present study, we knocked out the *PARP1* gene in the human HEK293FT line using a CRISPR/Cas9-based technique. This cell line is a fast-growing isolate derived from human embryonic kidney HEK293T cells [35]. The obtained cell clones with a putative *PARP1* knockout were characterized genetically, biochemically, and phenotypically using several approaches. The characterization included PCR analysis of deletions in genomic DNA, Sanger sequencing of genomic DNA, quantitative PCR (qPCR) analysis of mRNA encoding PARP1, Western blot analysis of WCE proteins with anti-PARP1 antibodies, and PAR synthesis in the WCEs.

Then, using qPCR in one clone, we assessed the levels of mRNAs coding for other BER-related proteins, namely PARP2, UNG2, APE1, POLβ, LIG3, and XRCC1, which participate in the SP pathway of BER. Simultaneously, we compared the efficiency of uracil removal, AP site cleavage, DNA synthesis, and ligation in the WCEs of the mutant cells and parental cells in the presence or absence of NAD^+^.

## 2. Materials and Methods

### 2.1. Materials

TEMED, bis-acrylamide, MgCl_2_, Tris, SDS, bromophenol blue, NaBH_4_, bovine serum albumin (BSA), dithiothreitol, acrylamide, ammonium persulfate, EDTA, and glycine were purchased from Sigma-Aldrich (St. Louis, MO, USA).

[γ-^32^P]ATP (5000 Ci/mmol) and [α-^32^P]ATP (3000 Ci/mmol) were produced in the Laboratory of Biotechnology, Institute of Chemical Biology and Fundamental Medicine SB RAS (ICBFM SB RAS; Russia). Synthetic oligonucleotides, including PCR primers, were obtained from Laboratory of Medicinal Chemistry, ICBFM SB RAS. The Master mix for RT-qPCR BioMaster RT-qPCR SYBR Blue (2×) and BioMaster HS-Taq PCR-Color (2×) for PCR were acquired from Biolabmix (Russia). Rabbit polyclonal antibodies to PARP1 were kindly provided by Dr. G.L. Dianov (Institute of Cytology and Genetics, SB RAS, Russia). Horseradish peroxidase (HRP)-conjugated secondary antibodies were obtained from the Laboratory of Biotechnology (ICBFM SB RAS).

Polynucleotide kinase of the T4 phage, *E. coli* UDG, and T4 DNA ligase were purified from *E. coli* cells overexpressing the corresponding proteins. The plasmids bearing cDNA of human AP endonuclease 1 and rat DNA polymerase β were kindly provided by Dr. S.H. Wilson (NIEHS, NIH, Bethesda, MD, USA). Recombinant proteins Polβ and APE1 were purified as described in refs. [36,37]. The vector coding for human PARP1 was a generous gift of Dr. V. Schreiber (ESBS, Illkirch, France). Recombinant PARP1 expressed in insect cells and purified as described in ref. [38] was a kind gift from Dr. M. Kutuzov (LBCE, ICBFM SB RAS).

The HEK293FT cell line was bought from Thermo Fisher Scientific (Waltham, MA, USA; cat. No. R70007). The laboratory stock of these cells was checked for the absence of mycoplasma contamination by PCR. Plasmid pSpCas9(BB)-2A-GFP (PX458) was a gift from Feng Zhang (Addgene plasmid #48138; http://n2t.net/addgene:48138; RRID: Addgene #48138). The pGEM-T Easy vector was purchased from Promega (Madison, WI, USA).

### 2.2. Cultivation of Cells

The growth medium for clone selection consisted of DMEM/F12 1:1 (Servicebio, Wuhan, China), 10% of fetal bovine serum (FBS), 100 U/mL penicillin–streptomycin, and 1× GlutaMAX (Thermo Fisher Scientific). HEK293FT WT and HEK293FT/P1-KO cells were grown in DMEM/F12 1:1 (Servicebio) in the presence of 10% of FBS (HyClone, Logan, UT, USA), 100 U/mL penicillin (Invitrogen, Carlsbad, CA, USA), and 100 µg/mL streptomycin (Thermo Fisher Scientific, Waltham, MA, USA) at 37 °C with 5% of CO_2_ in a humidified atmosphere.

### 2.3. Creation of PARP1 Knockout Cells (HEK293FT/P1-KO)

**Figure 1:**
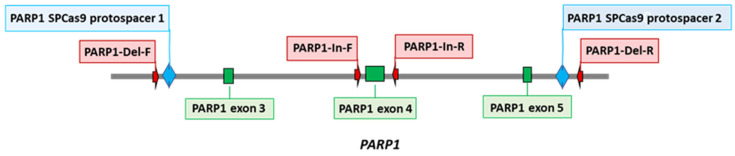
Positions of CRISPR/Cas9 protospacers and PCR primers used for the construction and characterization of HEK293FT/P1-KO cell clones.

PARP1 knockout HEK293FT clones were obtained from HEK293A cells as described previously [33]. Briefly, two protospacers for DNA sequence deletion that includes 3–5 exons of the *PARP1* gene (PARP1-gRNA1 and PARP1-gRNA2, Table S1) were selected using https://www.benchling.com/ (accessed on 9 November 2019). Positions of protospacers are shown in Figure 1. Corresponding oligonucleotides for expression of gRNA1 and gRNA2 were cloned into plasmid pSpCas9(BB)-2A-GFP (PX458). HEK293FT cells were transfected with plasmids PARP1-gRNA1 and PARP1-gRNA2 (0.25 µg each) using the Lipofectamine 3000 Reagent (Thermo Fisher Scientific, Waltham, MA, USA). Forty-eight hours after the transfection, a GFP-positive cell population was enriched via cell sorting using a BD FACS Aria III Cell Sorter (BD Biosciences, Franklin Lakes, NJ, USA). Single-cell clones grew for 2 weeks, and their genome was analyzed for CRISPR/Cas9-mediated deletions in the *PARP1* gene via PCR amplification of the target region. PCR was performed on DNA obtained by means of the QuickExtract™ DNA Extraction Solution (Lucigen, Madison, WI, USA) and the following primers: PARP1-Del-F and PARP1-Del-R for deletions and PARP1-In-F and PARP1-In-R for the wild-type allele (Table S1). The reactions were run on an S1000 Thermal Cycler (Bio-Rad, Singapore) using the following program: 95 °C for 3 min; then 35 cycles of 95 °C for 30 s, 60 °C for 30 s, and 72 °C for 30 s; followed by 72 °C for 3 min. The reaction products were resolved in a 1% agarose gel with ethidium bromide.

### 2.4. DNA Sequencing

To identify and map deletions in the *PARP1* gene, the PCR products obtained using primers PARP1-Del-F and PARP1-Del-R were cloned into the pGEM-T Easy vector (Promega). Ten independent plasmid clones were sequenced using the Sanger method with universal M13 primers at the SB RAS Genomics Core Facility (ICBFM SB RAS).

### 2.5. Estimation of Doubling Time

Doubling times for wild-type and mutant cells were determined in real time using an xCELLigence DP RealTime Cell Analyzer (ACEA Biosciences, Santa Clara, CA, USA). The cells were seeded in 16-well E-plates at a density of 10^5^ cells/well in 150 μL of the complete DMEM/F12 medium (Servicebio, Wuhan, China) supplemented with 1× GlutaMAX, 100 IU/mL penicillin, 100 µg/mL streptomycin (Thermo Fisher Scientific), and 10% of FBS (Thermo Fisher Scientific, Waltham, MA, USA) at 37 °C in a humidified atmosphere containing 5% of CO_2_ and were incubated for nearly 24 h to let the cells adhere to the bottom of the plate. The cells were incubated for the next 30 h under standard conditions. Cell index values were measured every 30 min.

### 2.6. Cell Culture Cytotoxicity Assay

Cytotoxicity of compounds was examined in HEK293FT WT and HEK293FT/P1-KO cells by the MTT test (Dia-m, Novosibirsk, Russia). Cells were seeded in 96-well plates (10,000 cells per well) in the DMEM/F12 medium (Servicebio) supplemented with 10% of FBS, penicillin (100 U/mL), and streptomycin (100 µg/mL) (Thermo Fisher Scientific) at 37 °C and 5% CO_2_ in a humidified atmosphere. The tested compounds were added to the medium at nearly 30% confluence. To determine the cytotoxicity of topotecan (Tpc, Actavis, Sindan Pharma, Romania) and temozolomide (Tmz, TCI Chemicals, Zwijndrecht, Belgium), the cells were incubated with one of the compounds at various concentrations for 72 h. All measurements were repeated at least twice.

### 2.7. ^32^P-Labeling of Oligonucleotides and Preparation of BER DNA Substrates

Oligodeoxyribonucleotides DNA-1 and DNA-2 (Table 1) were 5′-end-labeled with [^32^P]phosphate with T4 polynucleotide kinase and then purified with polyacrylamide gel (PAAG)/7 M urea electrophoresis according to ref. [39].

To obtain DNA duplexes, the complementary oligodeoxyribonucleotides were mixed in TE buffer (10 mM Tris-HCl pH 8.0 and 1 mM EDTA) at equimolar concentrations followed by heating at 97 °C for 5 min and slow cooling down to room temperature. The resulting DNA duplexes were analyzed via electrophoresis in a 10% PAAG under nondenaturing conditions.

### 2.8. Isolation of Total RNA

Total RNA was isolated using the TRIzol reagent (Thermo Fisher Scientific) according to ref. [40]. The purity of the isolated RNAs was accessed by means of the ratio of absorbance at 260 and 230 nm (A_260_/A_230_). A ratio of ~1.8–2.0 is generally assumed to indicate “purity” of RNA. For use in qPCR, the RNA was additionally treated with DNase to degrade possible traces of genomic DNA.

### 2.9. qPCR Analysis of mRNA Levels in HEK293FT WT and HEK293FT/P1-KO Cells

To estimate relative expression of genes encoding the key SP BER proteins in HEK293FT WT and HEK293FT/P1-KO cell lines, reverse-transcription qPCR (RT-qPCR) analysis was performed next. The reaction mixtures (20 μL) contained 0.5 ng/μL of total RNA, 0.5 µM primers, and 10 μL of BioMaster RT-qPCR SYBR Blue (2×). RT-qPCR was carried out on a LightCycler 96 system (Roche, Switzerland) under the following conditions: 1800 s reverse transcription at 45 °C, 300 s initial denaturation at 95 °C, 30 cycles of 10 s 95 °C denaturation, 10 s 58 °C primer annealing, and 10 s 72 °C primer elongation. Fluorescence was recorded during the annealing/elongation step in each cycle. A melting curve analysis was performed at the end of each PCR by gradually increasing the temperature from 58 to 95 °C and recording the fluorescence. Signal detection was carried out at 84 °C for 5 s. A single peak in the melting temperature curve of the amplicons confirmed specificity of the primers. The RT-qPCR was carried out in triplicate. Housekeeping genes *GAPDH*, *B2M*, *TUBΒ*, and *ACBT* served as calibrators. Primers were selected in the Primer-BLAST software (NCBI, Bethesda, MD, USA). The primers are listed in Table S1. For each pair of primers, the amplification efficiency was found to be in the range of 90–110%.

### 2.10. WCEs

WCEs from HEK293FT WT and HEK293FT/P1-KO cells were prepared as described elsewhere [41]. Protein concentration was measured by the Bradford assay [42]. The extracts were equalized by the concentration of total protein and stored in aliquots at −70 °C.

### 2.11. Western Blot Analysis of PARP1 in the WCEs of HEK293FT WT and HEK293FT/P1-KO Cells

This analysis was performed according to ref. [32]. In brief, WCE proteins (2.5 µg) or PARP1 (0.05 µg) were resolved by SDS 12.5% PAAG electrophoresis [43] followed by electrotransfer of proteins onto a nitrocellulose membrane using Trans-Blot Turbo (Bio-Rad, Hercules, CA, USA). The membrane was incubated in a solution of primary antibodies (rabbit antibodies to PARP1 at a dilution of 1:1000), then in a solution of secondary antibodies conjugated to HRP. The conjugate was stained using Super Signal West Pico PLUS (Thermo Fisher Scientific, Waltham, MA, USA). Chemiluminescence was detected on Amersham Imager 600 (GE Healthcare, Waukesha, WI, USA).

### 2.12. Synthesis of [^32^P]NAD^+^

The synthesis of radioactive NAD^+^ from [α-^32^P]-ATP was carried out as described before [44]. Briefly, reaction mixtures containing 1 mM ATP, 10 MBq of [α-^32^P]ATP, 20 mM MgCl_2_, 2 mM β-nicotinamide mononucleotide, and 5 mg/mL nicotinamide nucleotide adenylyltransferase in 25 mM Tris-HCl (pH 7.5) were incubated at 37 °C for 60 min, and the reactions were stopped by heating at 90 °C for 3 min. After removal of denatured proteins by centrifugation, the solution was used as a source of NAD^+^ without purification.

### 2.13. A PARP Activity Assay

The reaction mixtures (10 μL) were composed of 0.6 A_260_/mL of activated DNA, 20 μM [^32^P]NAD^+^, 5 mM MgCl_2_, 50 mM Tris-HCl (pH 8.0), 40 mM NaCl, 1 mM DTT, and 0.1 mg/mL BSA and were assembled on ice. WCE proteins at the final concentration of 0.5 mg/mL or 100 nM PARP1 were added as indicated in figure legends. The reaction mixtures were incubated at 37 °C for 1 min. The reaction was stopped by adding its aliquot of 4 μL dropwise on Whatman 1 paper filters pre-impregnated with trichloroacetic acid (TCA). PAR attached to proteins were precipitated on the filters in the presence of TCA. To remove unreacted NAD^+^, the filters were dried and washed three times with 150 mL of 5% ice-cold TCA, the rest of TCA was removed from paper using 90% ethanol, and the filters were dried and subjected to autoradiography for quantification.

### 2.14. Quantification of the Results of Autoradiography

After separation of the products, the PAAG gels were dried and then examined by autoradiography with the help of Biomolecular Imager Typhoon FLA 9500 (GE Healthcare). Radioactivity of the products was quantitated using the Quantity One software 4.6.8 (Bio-Rad).

### 2.15. Preparation of DNA Substrates Containing AP Sites or 5ʹ-dRP Residues

The uracil residue in the substrates was removed by means of *E. coli* UDG immediately before experiments. The reaction mixture consisted of 10 mM TE buffer pH 7.5, 1 μM uracil-containing DNA, and 0.1 U/μL UDG. The reaction was allowed to proceed at 37 °C for 20 min.

### 2.16. Tests for BER Enzymatic Activities in the WCEs

#### 2.16.1. An Assay of Uracil Excision Activity

The reaction mixtures (10 μL) were composed of the following components: 0.1 μM ^32^P-labeled uracil-containing DNA (Table 1, DNA-1 or DNA-3), either proteins of the WCE at the final concentration of 0.5 mg/mL or 0.1 U/μL *E. coli* UDG, 20 mM Tris-HCl (pH 8.0), 40 mM NaCl, 1 mM DTT, 5% of glycerol, 0.1% of Nonidet P-40, and 400 μM NAD^+^ (where specified). The reaction mixtures were incubated at 37 °C. Aliquots of 2 µL were transferred at 2, 5, and 10 min into the tubes that contained 2 µL of 100 mM NaOH with subsequent incubation at 60 °C for 10 min. Then, the reaction mixtures were supplemented with an equal volume of a mixture of 90% formamide and 10 mM EDTA and heated at 97 °C for 15 min followed by product separation in a 20% PAAG with 7 M urea and 10% of formamide [39]. The gels were dried and subjected to autoradiography.

#### 2.16.2. An Assay of AP Site Cleavage Activity

The reaction mixtures (10 µL) consisted of the following components: 0.1 µM ^32^P-labeled uracil-containing DNA duplex (DNA-3, Table 1), either WCE proteins at the final concentration of 0.01 mg/mL or 1 nM APE1, 5 mM MgCl_2_, and buffer components (10 mM Tris-HCl pH 8.0, 50 mM NaCl, 1 mM DTT, 5% of glycerol, 0.02% of Nonidet P-40, 0.1 mg/mL BSA, and 400 μM NAD^+^ [where specified]). AP sites in DNA were generated by UDG immediately before the assay of AP endonuclease activity. The reaction was carried out at 37 °C, and 2 μL aliquots were withdrawn at 2, 4, and 8 min; then, the reaction was stopped with an equal volume of a mixture of 100 mM methoxyamine and 50 mM EDTA, followed by incubation for 30 min at 0 °C. After that, the samples were supplemented with an equal volume of a mixture of 90% formamide and 10 mM EDTA and incubated at 0 °C for 30 min. The efficiency of AP site cleavage via the lyase mechanism was assayed similarly, except that the reaction mixture contained 20 mM EDTA instead of magnesium ions and incubation times were 10, 15, and 30 min. Further analysis was performed as described in the previous subsection.

#### 2.16.3. An Assay of DNA Polymerase Activity

The reaction mixtures (10 µL) contained 50 mM Tris-HCl (pH 8.0), 50 mM NaCl, 1 mM DTT, 0.1 mg/mL BSA, 5 mM MgCl_2_, 0.1 mM dNTPs, 100 nM 5′-^32^P-labeled DNA duplex (DNA-4, Table 1), 0.1% of Nonidet P-40, 5% of glycerol, either 0.5 mg/mL WCE proteins or 50 nM Polβ, and 400 μM NAD^+^ (where specified). Prior to DNA synthesis, DNA-5 was treated with UDG to obtain a DNA substrate (for SP BER) containing a 5′-dRP residue in the downstream oligonucleotide. The reaction mixtures were incubated at 37 °C. Aliquots of 2 µL were taken at 5, 10, and 15 min and supplemented with 1 μL of 25 mM EDTA to stop the reaction. Further analysis was performed as described above.

#### 2.16.4. An Assay of DNA Ligase Activity

The reaction mixtures (10 µL) were composed of buffer components (50 mM Tris-HCl pH 8.0, 50 mM NaCl, 1 mM DTT, 5% of glycerol, 0.1% of Nonidet P-40, and 0.1 mg/mL BSA), 0.1 μM ^32^P-labeled DNA duplex (DNA-5, Table 1), either 0.05 mg/mL WCE or 0.1 U/μL T4 DNA ligase, 5 mM MgCl_2_, 1 mM ATP, and 400 µM NAD^+^ (where specified). The mixtures were incubated at 37 °C. Aliquots of 2 µL were taken at 5, 15, and 30 min and mixed with 2 µL of a mixture of 90% formamide and 10 mM EDTA. Further analysis was performed as described above.

### 2.17. A Cell Culture Cytotoxicity Assay

Cytotoxicity of compounds was evaluated in HEK293FT WT and HEK293FT/P1-KO cells using the MTT test (Dia-m, Novosibirsk, Russia). Cells were seeded in 96-well plates (10,000 cells per well) in the DMEM/F12 medium (Servicebio) supplemented with 10% of FBS (Invitrogen), penicillin (100 U/mL), and streptomycin (100 µg/mL) at 37 °C and 5% CO_2_ in a humidified atmosphere. The tested compounds were added to the medium at nearly 30% confluence. To determine the cytotoxicity of topotecan (Tpc, Actavis, Sindan Pharma, Romania) and temozolomide (Tmz, TCI Chemicals, Zwijndrecht, Belgium), the cells were incubated with one of the compounds at the concentrations indicated in figure legends for 72 h. All measurements were repeated at least twice.

## 3. Results and Discussion

PARP1 is known as an abundant protein with many pleiotropic functions in cells. The involvement of PARP1 in the regulation of the BER process has been investigated in a wide range of experimental models, including purified proteins, cell extracts, cells, and organisms; each has its own advantages and disadvantages. One convenient and productive way to analyze the influences of PARP1 on the activity of DNA repair enzymes at each step of BER consists of a comparison of their activities in a reconstituted system containing a DNA intermediate with lesions specific for this step of DNA repair and WCEs from parental cells and cells devoid of PARP1. Short synthetic DNA duplexes containing specific lesions and WCEs have been used for a long time in the research into BER reactions and the influence of PARP1 on BER [44,45,46,47,48].

A systematic study elucidating how the absence of PARP1 affects the activities of key BER proteins and levels of their mRNAs in human cells has not yet been conducted. The use of human cells with a complete absence of PARP1 opens up new possibilities to study the role of this protein in the BER system at the level of the proteins themselves and/or of their activities as well as at the level of the mRNAs encoding them.

### 3.1. Generation and Characterization of HEK293FT/P1-KO Cells

In spite of the attractiveness of model cells with the *PARP1* gene knockout for various studies, only a limited number of such human cell lines created by CRISPR/Cas9 [33,49,50,51,52] or TALEN [53,54,55] gene editing are known at present.

Here, *PARP1* knockout HEK293FT clones (HEK293FT/P1-KO) were generated essentially as previously described for *PARP1* knockout HEK293A cells [33]. The positions of protospacers for the deletion of a DNA sequence including 3–5 exons of the *PARP1* gene, which encode a part of the DNA-binding domain, as well as primer sequences for the detection of deletions are shown in the schema (Figure 1).

**Figure 2:**
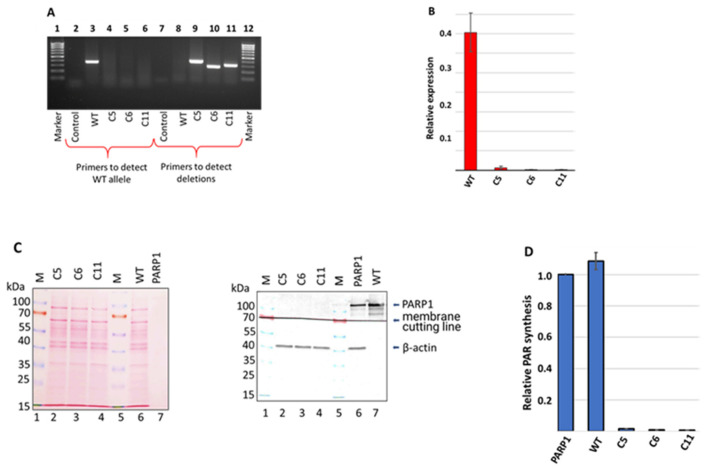
Analysis of the PARP1 content of HEK293FT WT and HEK293FT/P1-KO clones. (**A**) PCR analysis of genomic DNA of selected clones. Lanes 1 and 12: a 100–1000 bp DNA ladder; lanes 2–6: products of PCR with primers for detection of the *PARP1* wild-type allele; lanes 7–11: products of PCR with primers for the detection of the deletion in the *PARP1* gene. (**B**) Quantification of *PARP1* mRNA in the cells. The results are shown as mean ± standard deviation as obtained in three independent experiments. (**C**) Western blot analysis of the PARP1 protein in WCEs of the clones. β-Actin served as an internal control of loading. The left panel shows Ponceau S staining of the membrane. Right panel: Antibody detection of PARP1 and β-actin. (**D**) Efficiency of PAR synthesis in the WCEs. The relative level of PAR synthesized by endogenous PARPs for 1 min at 37 °C is presented. In each experiment, the amount of PAR synthesized by the extract PARPs was normalized to that synthesized by 100 nM recombinant PARP1. The data are expressed as the mean ± standard deviation of three independent experiments.

Approximately 1 week after the transfection of HEK293FT WT cells with corresponding plasmids, individual clones were picked for genotype analysis around the gRNA-targeted site through a PCR analysis of the genomic DNA. Three clones, designated as C5, C6, and C11, in which the PCR analysis did not detect a product characteristic for the wild-type gene (Figure 2A, lanes 3–6), were selected for subsequent propagation and experiments.

The data of the PCR analysis with specific primers confirmed the presence of intended deletions in clones C5, C6, and C11 (Figure 2A, lanes 8–11). The genomic DNA of the three selected cell clones (C5, C6, and C11) was sequenced via the Sanger method. Data are shown in Figure S1. In all three clones, the analysis revealed extended deletions differing in positions and length. The sequencing data indicated that in each clone, only one type of deletion was generated, which may be explained, for instance, by the presence of a single allele coding for PARP1 in the cell genome. Indeed, it has been reported [56] that HEK293 cells, i.e., the progenitor cell line of HEK293FT cells, contain only one copy of chromosome 1 harboring the *PARP1* gene, whereas in another study [57], three copies were found.

After confirming the deletions in the *PARP1* gene at the genomic level, we evaluated, using qPCR, the level of mRNA coding for PARP1 (Figure 2B and Figure S2). No appreciable amount of the mRNA-encoding PARP1 was detected in all three HEK293FT/P1-KO cell clones.

Next, we confirmed the *PARP1* gene knockout at the protein level via a Western blot analysis. The analysis of the WCEs of the HEK293FT/P1-KO clones and the HEK293FT WT cells did not reveal a detectable amount of PARP1 in mutant cells (Figure 2C, compare lanes 2–4 and lane 6).

To further characterize the mutant cell lines, the efficiency of poly(ADP) ribose (PAR) synthesis, catalyzed by endogenous PARPs of WCEs, was estimated. ^32^P-Labeled NAD^+^ and activated DNA as a cofactor were used (Figure 2D). We chose the “1 min” time point, which is in the linear part of the kinetic curve of PAR synthesis by endogenous PARPs of the WCEs, judging by a preliminary analysis. In each experiment, the amount of PAR synthesized in the cell extracts was normalized to that synthesized by 100 nM recombinant PARP1. As expected, almost no synthesis of PAR was detectable in the extracts of HEK293FT/P1-KO cells. Overall, these results are in full agreement with the known main contribution of PARP1 to PAR synthesis in the cell [3,4,5,6,7,8,9,10,11,12]. Thus, the obtained three clones of HEK293FT/P1-KO cells do not express a detectable amount of PARP1.

The doubling time of the HEK293FT/P1-KO clones and HEK293FT WT cells was estimated in real time using the xCELLigence device. Data are presented in Figure S2. The cells’ doubling time was 9.6 ± 0.1 h for the WT, 9.3 ± 0.3 h for clone C5, 10.0 ± 0.1 h for C6, and 13.2 ± 0.3 h for C11.

Data about the effects of topotecan and temozolomide on cell viability in the MTT assay are shown in Figure S3. PARP1 knockout cells were somewhat more sensitive to topotecan than HEK293FT WT cells. Our obtained HEK293FT/P1-KO clones did not have a significant difference in sensitivity to temozolomide as compared with the parental cells.

Cell clone C5 was selected as representative HEK293FT/P1-KO cells for the subsequent biochemical characterization of PARP1′s influence on the BER system. All the data below mentioning HEK293FT/P1-KO cells refer to clone C5.

### 3.2. The Comparison of PARP2, UNG2, APEX1, POLΒ, and XRCC1 mRNA Relative Expression Levels

Taking into account the known participation of PARP1 in the regulation of pre-mRNA splicing, in the maintenance of mRNA stability/decay [13], and in transcriptional regulation [14], we intended to evaluate the impact of PARP1′s full absence in the cells on the levels of mRNAs encoding the proteins taking part in the BER process. To this end, using samples of RNA obtained from HEK293FT WT and HEK293FT/P1-KO cells (clone C5), we compared relative amounts of mRNAs coding for PARP2, UNG2, APE1 (Apex1), DNA polymerase β (Polβ), DNA ligase III (LIG3), and XRCC1 in the above cells using qPCR.

**Figure 3:**
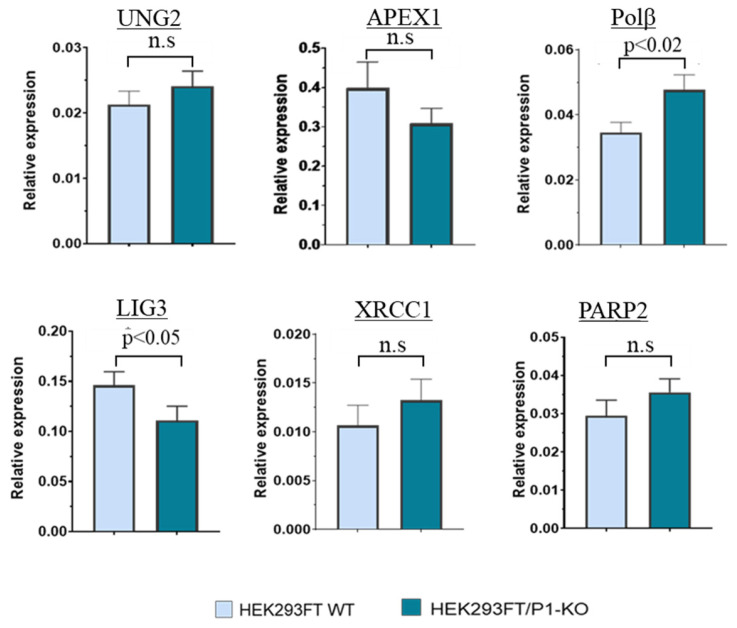
qPCR analysis of relative expression of mRNAs coding for the key SP BER proteins in HEK293FT WT and HEK293FT/P1-KO cells: UNG2, APEX1, Polβ, XRCC1, LIG3, and PARP2. The data are expressed as the mean ± standard deviation of at least three independent experiments; n.s.: not significant.

Amplification efficiency was estimated for each primer pair, and these values were in the range of 90–110%. Examples of PCR product accumulation for LIG3 and Polβ are presented in Figure S4. Summarized data on mRNA relative expression of SP BER proteins are given in Figure 3.

Overall, no statistically significant differences in the mRNA levels of UNG2, APEX1, XRCC1, and PARP2 were found, in contrast to Polβ and LIG3, for which a less than 1.5-fold difference (but statistically significant) was revealed. For Polβ, the increase in the mRNA level in P1-KO cells was about 1.4-fold. Unlike the current study, a transcriptomic analysis of HEK293A PARP1 knockout cells has revealed a ~1.5-fold downregulation of Polβ mRNA [34].

### 3.3. The Influence of PARP1 Presence in WCEs on the Efficiency of BER Reactions

To assess the impact of PARP1 on the efficiency of the SP BER reactions catalyzed by endogenous enzymes, we compared the enzymatic activities using functional assays: excision of uracil, AP site processing, DNA synthesis, and DNA ligation. In the functional assays, ^32^P-labeled DNA structures imitating intermediates of a given stage of BER were used together with WCEs from HEK293FT WT and HEK293FT/P1-KO cells. Examples of electrophoretic analysis of the products of the BER reactions are shown in Figure S4. To evaluate the impact of PARylation, all functional assays were performed in parallel in the absence and presence of NAD^+^. It should be noted that when the effect of PAR synthesis on the activity of endogenous enzymes was determined in the extracts, a DNA substrate characteristic for a certain stage of BER acted as a cofactor DNA, which is necessary for PARP1 activation. A comparison of the cofactor characteristics of the activated DNA traditionally used in assays of PARP1 activity and the 0.1 μM BER DNA substrates employed in the tests of the BER enzymatic activities revealed comparable levels of PARP1 activation (Figure S6).

#### Uracil Removal

Uracil residues in genomic DNA may emerge due to the occasional substitution of thymine during DNA synthesis or as a consequence of spontaneous and enzymatic cytosine deamination. It is thought that 70–200 spontaneous cytosine deamination events occur in a single cell daily [58]. Three types of uracil-DNA glycosylases have been found in higher eukaryotic cells [59]: single strand-selective monofunctional uracil glycosylase (SMUG), mitochondrial UNG1, and nuclear UNG2. Uracil N-glycosylases UNG2 and SMUG1 initiate error-free BER in most DNA contexts [59].

No statistically significant difference in the mRNA level of UNG2 between HEK293FT WT and HEK293FT/P1-KO cells was detected using qPCR. Of note, in HEK293A cells, PARP1 knockout leads—as determined by transcriptomic analysis—to the under expression of four DNA glycosylases, with another uracil DNA glycosylase, SMUG1, being among them [34].

We compared the efficiency of uracil removal from double-stranded and single-stranded DNAs (DNA-1 and DNA-3, Table 1). To track the uracil excision, a DNA intermediate was subjected to alkaline treatment, which splits sugar-phosphate backbone at the position of an AP site arising after uracil removal. Examples of the electrophoretic analysis of products of uracil excision by endogenous uracil DNA glycosylases from WCEs on single-strand and double-strand substrates are shown in Figure S5A.

**Figure 4:**
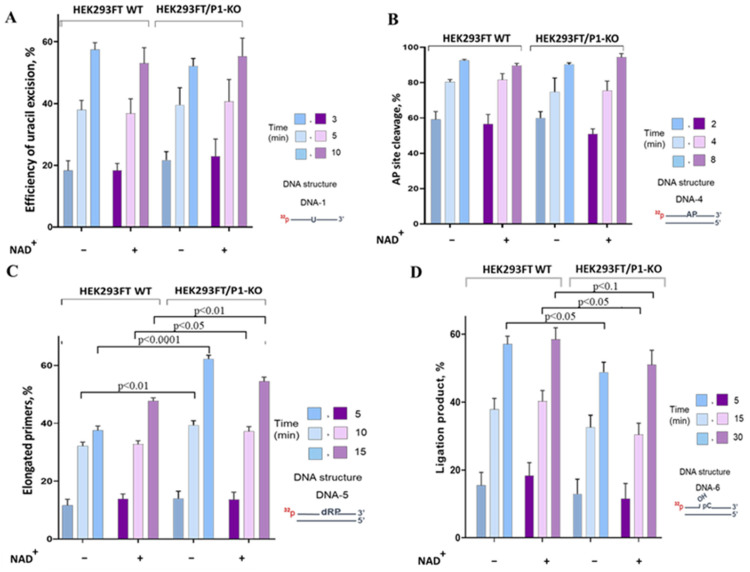
Efficiency of the BER reactions catalyzed by endogenous enzymes from the WCEs of HEK293FT WT and HEK293FT/P1-KO cells. (**A**): uracil excision from single-stranded substrate DNA; (**B**): cleavage of an AP site; (**C**): elongation of a primer; (**D**): ligation of a nick. The data are expressed as the mean ± standard deviation of at least three independent experiments.

Summarized data on uracil excision by the WCE uracil DNA glycosylases in the presence and absence of NAD^+^ are given in Figure 4A.

The efficiency of uracil excision by the WCE uracil DNA glycosylases was ~10% higher on single-stranded DNA compared with double-stranded DNA (Figure S5A). This efficiency was not influenced by the presence or absence of PARP1. Additionally, the presence of NAD^+^ in the reaction mixtures did not affect the efficiency of uracil removal (Figure 4A). Therefore, there was no evidence of PARP1′s influencing uracil excision in the WCEs.

The next step of SP BER is the processing of the AP site. This site can be cleaved in several ways: via hydrolysis catalyzed by specific AP endonucleases 1 and 2 and tyrosyl-DNA phosphodiesterase 1 or through an AP-lyase-dependent splitting mechanism [60]. The hydrolysis is known to be the predominant route of AP site processing in mammalian cells [61], with APE1 being the enzyme responsible for cleavage in the majority of AP sites [62]. Our previous study about PARP1–APE1 interplay on an earlier BER DNA intermediate (an AP site-containing DNA) in a system reconstituted from individual proteins has revealed the inhibition of APE1 activity by PARP1, both on naked DNA and in the context of reconstituted nucleosome core particles [30,31,48]. The auto-PARylation of PARP1 in the presence of NAD^+^ resulted in a decrease in its inhibitory effect.

To analyze the processing of an AP site, uracil-containing double-stranded DNA (Table 1, DNA-3) was converted to an AP site-containing substrate immediately prior to the experiment through treatment with *E. coli* uracil DNA glycosylase. Examples of electrophoretic analysis of the products arising after AP site cleavage by endogenous enzymes of WCEs are displayed in Figure S5B. The summarized data of AP site cleavage by the WCE enzymes in the presence or absence of NAD^+^ are presented in Figure 4B. AP site processing by the extracts of HEK293FT cells did not differ between WT and P1/KO cells, although the APE1 mRNA level was slightly higher in WT cells (Figure 3). The presence of NAD^+^ did not interfere with AP site processing in WCEs of mutant and parental cells.

The APE1-independent activity was estimated by testing AP site cleavage in the presence of EDTA, to bind Mg^2+^ ions, which are absolutely required for APE1 functioning [61,62]. The efficiency of AP site cleavage was ~4–6%.

DNA synthesis in DNA with a gap formed after AP site processing is the next BER step, which is conducted by DNA polymerases, with Polβ being the main DNA polymerase of SP BER [15,16,17,18,19,20,21,22]. Prior studies by our laboratory indicate that in a system reconstructed from purified PARP1 and Polβ, PARP1 (which possesses strong affinity for DNA -containing single-strand breaks) can inhibit Polβ activity, but the inhibitory effect is more pronounced on a substrate of LP BER than on an SP BER substrate [31].

Here, we compared DNA polymerase activity between WCEs of HEK293FT WT and HEK293FT/P1-KO cells (Figure 4C and Figure S5C). The findings suggested that the PARP1 deletion in cells leads to a 10–15% increase in primer elongation by the extract enzymes. A slight increase in the level of the mRNA-encoding Polβ was observed in HEK293FT/P1-KO cells (Figure 3); this phenomenon can potentially result in a higher concentration of the enzyme in this extract. Thus, the observed increase in the primer elongation may be caused by a lack of competition between Polβ and PARP1. When NAD^+^ was present, no significant influence of PARylation on DNA synthesis was observed either in the HEK293FT WT WCE or in the HEK293FT/P1-KO WCE. There was a weak influence of PARylation on DNA synthesis catalyzed on the DNA intermediates of SP and LP BER pathways by endogenous DNA polymerases of WCEs obtained from mouse fibroblasts or naked mole rat fibroblasts or HEK293T cells [44]. It should be pointed out that the WCEs of these cells manifested a striking difference in the efficiency of PAR synthesis while the efficiency of DNA synthesis was comparable among all WCEs.

The last SP BER step—ligation—is conducted by ATP-dependent DNA ligase 3α (LIG3). LIG3 forms a stable functionally active complex with the structural XRCC1 protein [63,64]. XRCC1 has no catalytic activity but forms binary and ternary complexes with several BER enzymes (proteins) to ensure their concerted action, which to a substantial extent, is determined by XRCC1′s abilities to bind BER proteins, to undergo PARylation, and to bind PAR [2,63,65].

We estimated DNA ligase activity in the WCEs using nicked DNA (DNA-5, Table 1). A weak but statistically significant decrease in the efficiency of DNA ligation was observed in the WCE of HEK293FT/P1-KO cells (Figure 4D). The lower efficiency of ligation could be related to the observed downregulation of mRNA coding for LIG3 (Figure 3). Again, there was no influence of NAD^+^ presence regardless of the extract source: WT or PARP1 knockout cells.

## 4. Conclusions

The roles of DNA-activated PARPs, such as PARP1 and PARP2, in BER are still being actively investigated. A negative impact of PARP1 and PARP2 on the BER reactions caused by their competition with the BER enzymes for the same DNA intermediates can be overcome through PARylation [30,31,48]. On the other hand, it has been suggested that PAR may play a role in the formation of cellular compartments by interacting with BER proteins to concentrate them on DNA damage [2]. Additionally, the participation of PARP2 in the regulation of BER should not be underestimated, especially considering the discovery of such proteins as histone PARylation factor 1 (HPF1), which can considerably enhance the activity of PARP2 until the level demonstrated by PARP1 on intermediate of BER [66]. This phenomenon may allow PARP2 to substitute for PARP1 in certain processes.

It has been recently demonstrated that in the system reconstituted from recombinant proteins (PARP1, APE1, Polβ, LIG3, and XRCC1), PARylation causes a decrease in the BER efficiency, which appears to be due to the lowered affinity of the PARylated proteins to DNA substrates [67]. The decrease in affinity resulting in the easier dissociation of the protein-DNA complexes suggests a “contribution of PARylation to the active turnover of the BER complex”.

Notably, the absence of PARP1 in cells does not significantly affect the mRNA levels of BER enzymes or the activities of the main SP BER enzymes. It appears that the relatively weak effects of PARP1 may contribute to the fine-tuning of the activities of BER enzymes in the absence of severe cell stress. This idea is consistent with the current notion that the BER system has redundant capacity, and excess unused proteins are deactivated, for example, through the ubiquitin-dependent proteolysis system [68,69,70].

Our results are in full agreement with the viability of PARP1 knockout cells in the absence of genotoxic stress, which was demonstrated both for mouse and human models [25,26,27,28,29,33,34,49,50,51,52,53,54,55].

## Figures and Tables

**Table 1:** DNA used in the functional assays of the BER enzymatic activities.

Name	Sequence	Structure
DNA-1	5′-pGGGAGGCCCTGGCGTT**U**CCCGGCTTAGTCGCC-3′	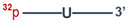
DNA-2	5′-pGGCGACTAAGCCGGG-3′	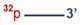
DNA-3	5′-pGGGAGGCCCTGGCGTT**U**CCCGGCTTAGTCGCC 3′-CCCTCCGGGACCGCAAGGGGCCGAATCAGCGG	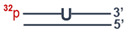
DNA-4	5′-pGGCGACTAAGCCGGG p**U**CCCGGCTTAGTCGCC 3′-CCGCTGATTCGGCCCGTTGCGGTCCCGGGCGG	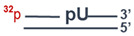
DNA-5	5′-pGGCGACTAAGCCGGG pCAACGCCAGGGCCTCCC 3′-CCGCTGATTCGGCCCGTTGCGGTCCCGGAGGG	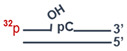

## Data Availability

Data is contained within the article and Supplementary Material.

## Supplementary Materials

The following supporting information can be downloaded at: https://www.mdpi.com/article/10.3390/cells13040302/s1. Table S1. Oligonucleotide sequences used in creation of PARP1 knockout cells. Table S2. Primers used in qPCR. Figure S1. Alignment of the DNA sequence of the wild-type PARP1 gene and sequencing of the plasmid containing the PCR products, which were obtained from the genomic DNA of HEK293FT clones C5 (A), C6 (B) and C11 (C) with putative deletions in the PARP1 gene. Only for three sequenograms for each clone are shown. Figure S2. Estimation of doubling time for HEK293FT WT and 3FT/P1-KO cells. Figure S3. Sensitivity of HEK293FT WT and 3FT/P1-KO cells to topotecan and temozolomide. Figure S4. qRT-PCR analysis of Pol beta and Lig3 mRNAs in HEK293FT/P1-KO clone C5 and HEK293FT WT cells. Figure S5. Efficiencies of the BER reactions catalyzed by endogenous enzymes of the WCEs of HEK293FT WT and HEK293FT/P1-KO cells. A—uracil excision from single-strand and double-strand substrate DNAs. Time: 3, 5, 10 min; B—cleavage of AP site. Time: 2, 4, 8 min; C—elongation of the primer. Time: 5, 10, 15 min; D—ligation of the nick. Time: 5, 15, 30 min. Figure S6. Comparison of PARP1 activation by different DNAs. SSDNA-DNA—Non-labeled DNA-1, DS—Non-labeled DNA-3 (Table 1).
