# Supplementary material for: A Knockout of Poly(ADP-Ribose) Polymerase 1 in a Human Cell Line: An Influence on Base Excision Repair Reactions in Cellular Extracts
Source: Cells. 2024 Feb 6;13(4):302. doi: 10.3390/cells13040302 (PMC10886765; doi:10.3390/cells13040302)
Supplement: Supplementary file 1 [file cells-13-00302-s001.zip › cells-2777676-supplementary.pdf]

A Knockout of Poly(ADP-Ribose) Polymerase 1 in a Human Cell Line: An Influence on Base Excision Repair Reactions in Cellular Extracts

[Дата]

Table S1. Oligonucleotide sequences used in creation of PARP1 knockout cells

Name	Sequence	Function
PARP1-gRNA1	5′-CTAGAACCTCCAATACCATG (TGG)	Protospacer
PARP1-gRNA2	5′-GCAAGTGACCACAAAGGTGC (AGG)	Protospacer
PARP1-Del-F	5′-AGTGTGCCCTGCGTATTTGC-3′	Forward primer for detection of deletion
PARP1-Del-R	5′-CGCTCCCTTGGTACCACATATG-3′	Reverse primer for detection of deletion
PARP1-In-F	5′-CGCTCCCTTGGTACCACATATG-3′	Forward primer for detection of WT allele
PARP1-In-R	5′-GGCTTACTGACAGTCAGCGAAG-3′	Reverse primer for detection of WT allele

Table S2. Primers used in qPCR

Gene	Sequences of forward/reverse primers
Gapdh	5′-AGATCATCAGCAATGCCTCCT-3′/ 5′-TGGTCATGAGTCCTTCCACG-3′
B2M	5′-CGCTCCGTGGCCTTAGCTGT-3′/ 5′-AAAGACAAGTCTGAATGCTC-3′
Tubβ	5′-TGGTGGATCTAGAACCTGGGA-3′/ 5′-CTGCCCCAGACTGACCAAAT-3′
ACBT	5′-TTCCTGGGCATGGAGTCCT-3′/ 5′-TGTGTTGGCGTACAGGTCTT-3′
Parp1	5′-GCTTCAGCCTCCTTGCTACAG-3′/ 5′-CTTCTTCGCCACTTCATCCAC-3′
Parp2	5′-TCCTAAGGCCGAAGGATTGC-3′/ 5′-CCCATTCAGGGTGACGAAGT-3′
Ung2	5′-AAGCAAGGTGTTCTCCTTCTCA-3′/ 5′-GCCAGGACACAACTGCATC-3′
Apex1	5′-GATCTCGCGAGTAGGGCAAC-3′/ 5′-TTCGGCATTCCCGTTACGAA-3′
Polβ	5′-GAACACTCTGGGGTTCTCGG-3′/ 5′-TGCGAGTTCTGTGAGCATGT-3′
Lig3	5′-CCAGGCTAAGTTGACAACCAC-3′/ 5′-TGTTGGGCTTGGCTGAAAAG-3′
Xrcc1	5′-TACAGCAAGGACTCCCCCTT-3′/ 5′-CACTGTCACCTTCTGGGACG-3′

[Дата]

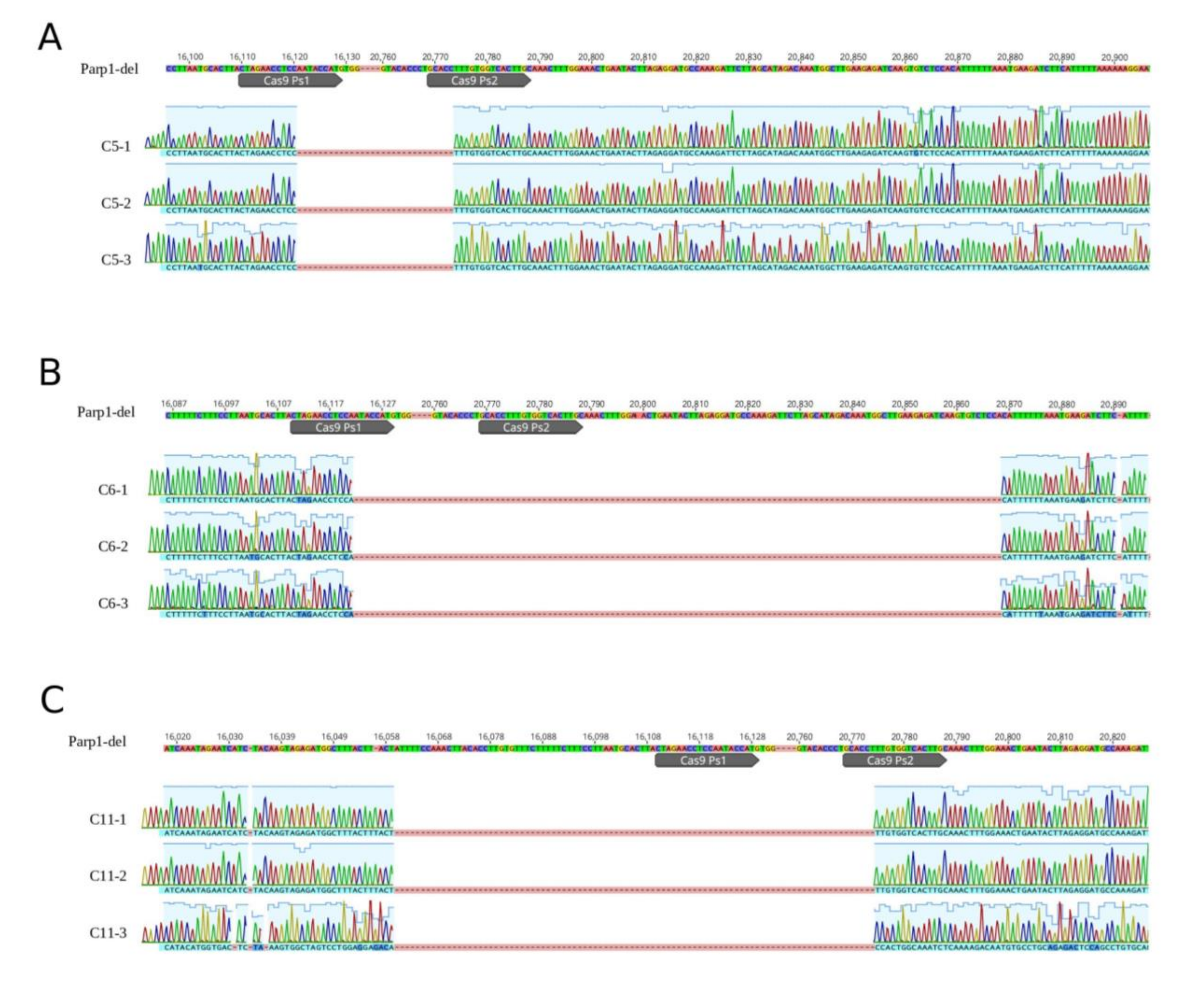

Figure S1. Alignment of the DNA sequence of the wild-type PARP1 gene and sequencing of the plasmid containing the PCR products, which were obtained from the genomic DNA of HEK293FT clones C5 (A), C6 (B) and C11 (C) with putative deletions in the PARP1 gene. Only for three sequenograms for each clone are shown.

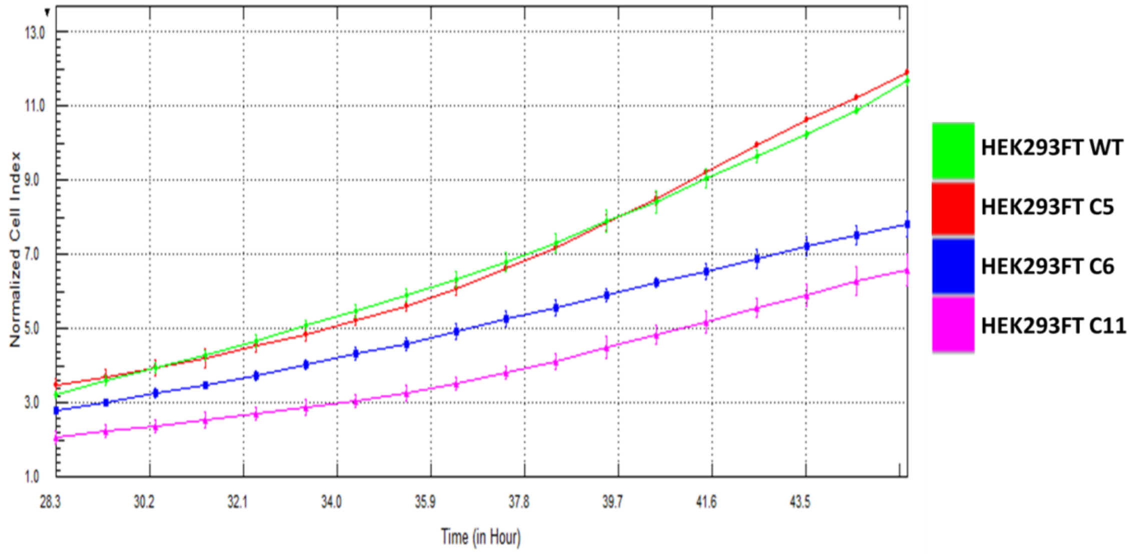

Figure S2. Estimation of doubling time for HEK293FT WT and 3FT/P1-KO cells.

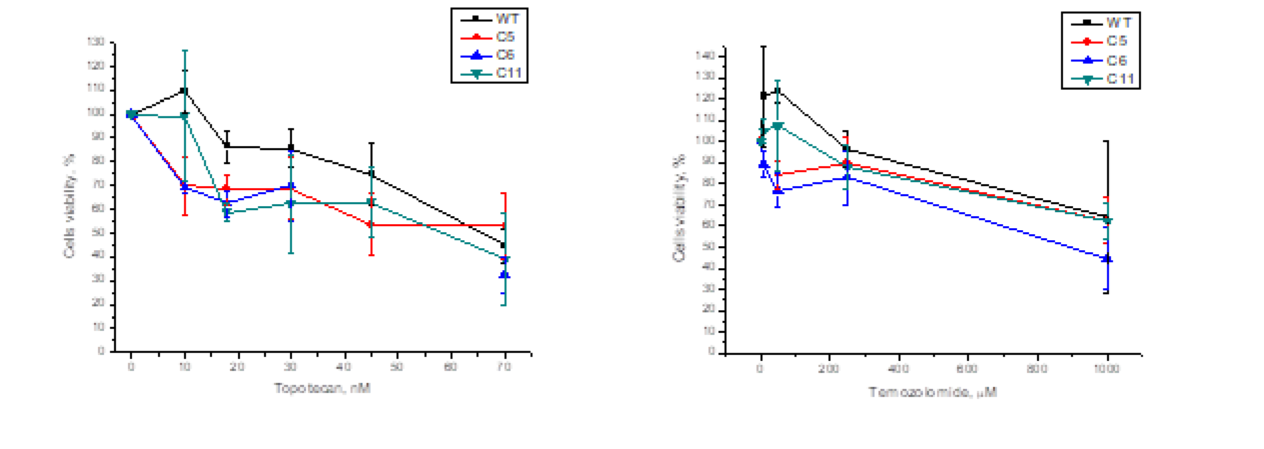

Figure S3. Sensitivity of HEK293FT WT and 3FT/P1-KO cells to topotecan and temozolomide.

Sample Name	Gene Name	Ratio	Ratio Error	Cq Mean	Cq Error
HEK293FT/P1-KO	Lig3	1.028E+0	7.570E-2	19.85	0.06
HEK293FT WT	Lig3	1.467E+0	7.608E-2	19.12	0.02

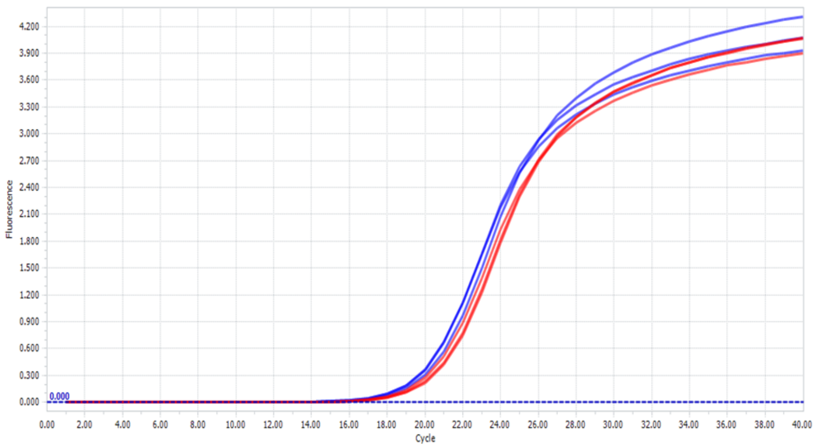

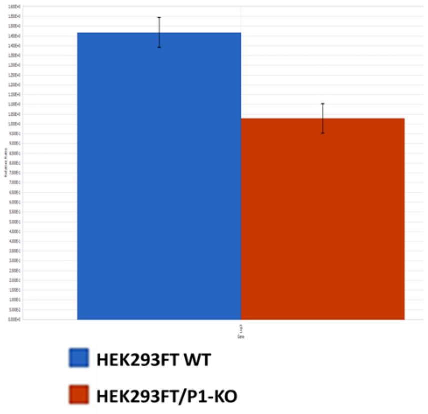

Sample Name	Gene Name	Ratio	Ratio Error	Cq Mean	Cq Error
HEK293FT WT	Polβ	3.456E-2	3.119E-3	23.95	0.01
HEK293FT/P1-KO	Polβ	4.762E-2	4.664E-3	23.29	0.10

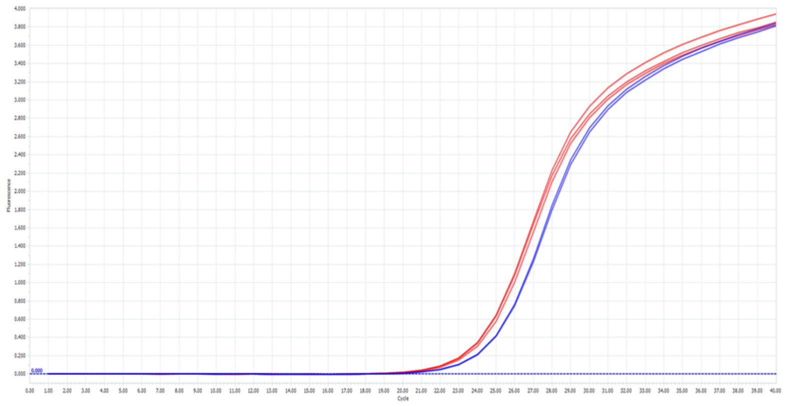

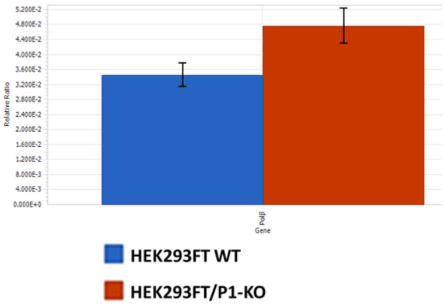

Figure S4. qRT-PCR analysis of Pol beta and Lig3 mRNAs in HEK293FT/P1-KO clone C5 and HEK293FT WT cells.

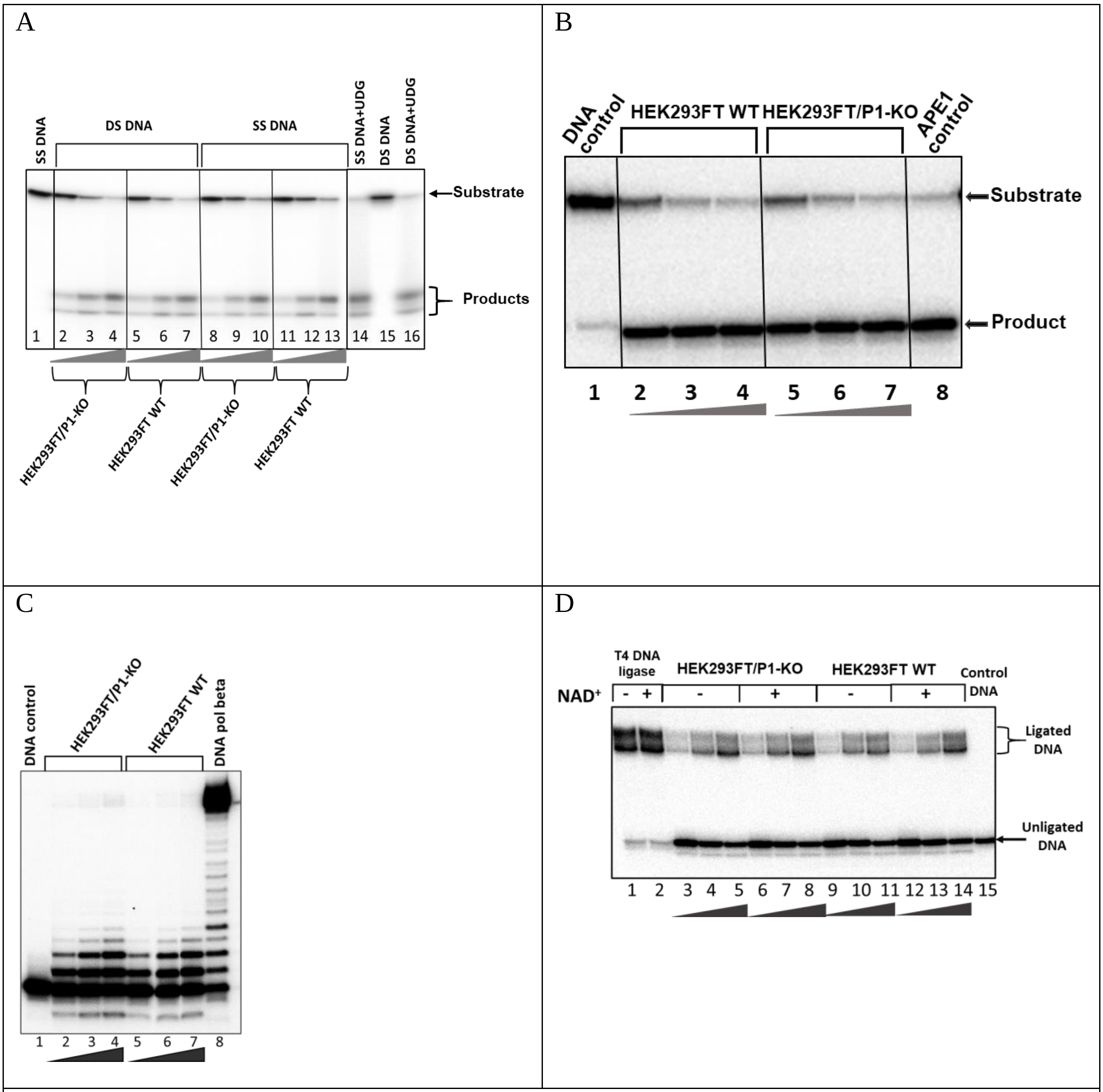

Figure S5. Efficiencies of the BER reactions catalyzed by endogenous enzymes of the WCEs of HEK293FT WT and HEK293FT/P1-KO cells. A – uracil excision from single-strand and double-strand substrate DNAs. Time: 3, 5, 10 min.; B – cleavage of AP site. Time: 2, 4, 8 min.; C – elongation of the primer. Time: 5, 10, 15 min.; D – ligation of the nick. Time: 5, 15, 30 min.

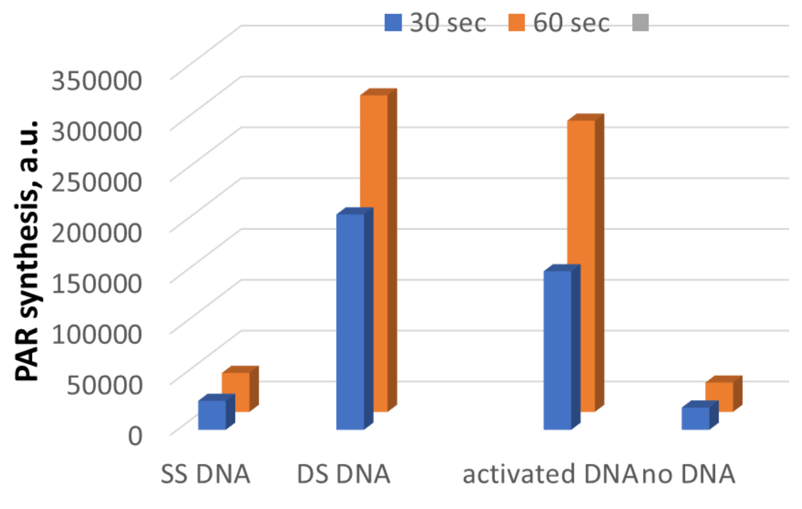

Figure S6. Comparison of PARP1 activation by different DNAs.

SSDNA-DNA – Non-labeled DNA-1, DS - Non-labeled DNA-3 (Table 1).
